# Myelodysplastic Syndrome with Myelofibrosis Transformed to a Precursor B-Cell Acute Lymphoblastic Leukemia: A Case Report with Review of the Literature

**DOI:** 10.1155/2012/207537

**Published:** 2012-03-29

**Authors:** Ayed A. Algarni, Mojtaba Akhtari, Kai Fu

**Affiliations:** ^1^Department of Pathology and Microbiology, University of Nebraska Medical Center, Omaha, NE 68198, USA; ^2^Department of Internal Medicine, University of Nebraska Medical Center, Omaha, NE 68198, USA

## Abstract

Myelodysplastic syndromes (MDS) comprise a group of heterogeneous clonal hematopoietic cell disorders characterized by cytopenias, bone marrow hypercellularity, and increased risk of transformation to acute leukemias. MDS usually transformed to acute myeloid leukemia, and transformation to acute lymphoblastic leukemia (ALL) is rare. Herein, we report a unique patient who presented with MDS with myelofibrosis. Two months after the initial diagnosis, she progressed to a precursor B-cell acute lymphoblastic leukemia. She was treated with induction therapy followed by allogenic stem cell transplantation. She was alive and doing well upon last followup. We have also reviewed the literature and discussed the clinicopathologic features of 36 MDS patients who progressed to ALL reported in the literature.

## 1. Introduction

Myelodysplastic syndrome (MDS) comprises a group of heterogeneous clonal hematopoietic cell disorders characterized by cytopenias, bone marrow hypercellularity, and increased risk of transformation to acute leukemias; usually to acute myeloid leukemia (in 20–50% of MDS patients). Transformation of MDS to acute lymphoblastic leukemia (ALL) is rare. To date, there are only 35 reported cases of MDS progressed to ALL, to the best of our knowledge. Herein, we report a unique patient who presented with MDS and myelofibrosis and progressed to precursor B-cell acute lymphoblastic leukemia shortly after the initial presentation.

## 2. Case Report

A 53-year-old Caucasian woman with a known history of diabetes mellitus type 2, osteoarthritis, hypertension, and hyperlipidemia presented, after a possible acute upper respiratory tract infection, with shortness of breath, fatigue, nausea, and vomiting. Initial physical examination showed no lymphadenopathy or hepatosplenomegaly. Her CBC revealed pancytopenia with white blood cell (WBC) count = 1.8 × 10^9^/L, hemoglobin = 4.5 g/dL, mean corpuscular volume (MCV) = 84.8 fL, hematocrit = 12.5, and platelet count = 83 × 10^3^/cmm.

**Figure 1:**
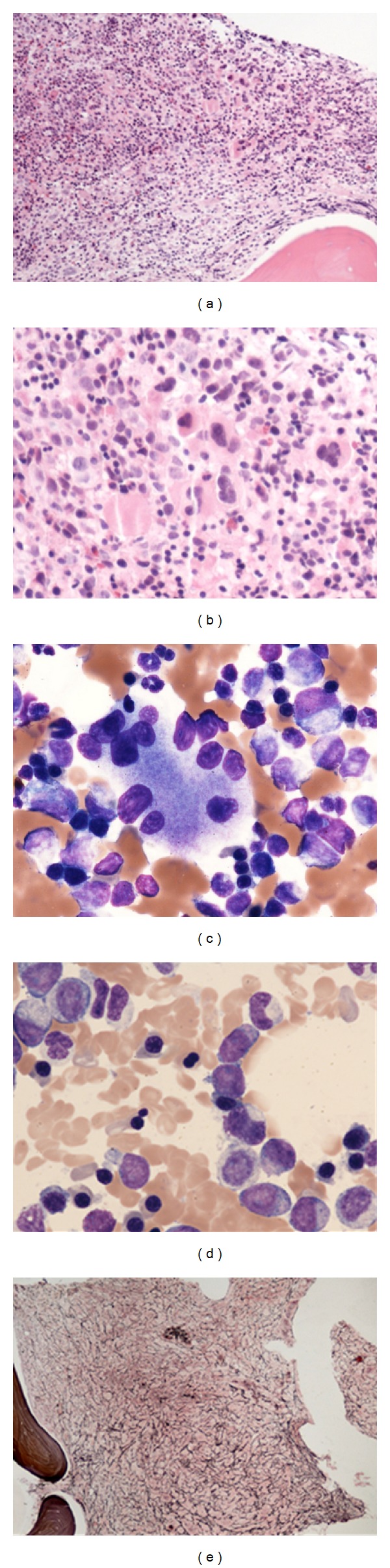
The initial bone marrow biopsy showing panhyperplasia with focal clustering of megakaryocytes (a) and (b) as well as dyspoietic multinucleated megakaryocytes (c) and dysplastic erythroid precursors (d) are seen. Severe reticulin fibrosis in the initial biopsy was also noted (e).

She underwent a bone marrow aspirate and biopsy. The bone marrow aspiration was unsuccessful. The bone marrow biopsy showed a markedly hypercellular bone marrow at 90% cellularity with panhyperplasia (Figure 1). Mild-to-moderate erythroid dysplasia was present in the form of binucleation, nuclear blebbing and irregular nuclear contours. The megakaryocytes were moderately increased with focal abnormal clustering and occasional dyspoietic forms like nuclear hypolobation and micromegakaryocytes. The myeloid precursors showed abnormal localization in the core biopsy without significant morphological dyspoiesis. Blasts were not increased at 0.4%, with only rare scattered CD34-positive cells in the core biopsy by immunostains. Scattered clusters of small lymphocytes with occasional irregular nuclear contours were present; however, no clusters of lymphoblasts are noted. Special stains for reticulin and collagen fibers showed marked reticulin fibrosis without significant collagenous fibrosis. Conventional cytogenetic studies showed a normal female chromosome karyotype. Florescence in situ hybridization (FISH) studies using probes for MDS including MLL gene (11q23) region, monosomy 7, and trisomy 8 and for deletions of 5q31, 7q31 and 20q12 were performed and were negative. Molecular studies for BCR-ABL and JAK2 mutation studies were also negative. A diagnosis of myelodysplastic syndrome (MDS) with myelofibrosis was rendered. Regarding the MDS International Prognostic Scoring Classification [26], the patient had 3 cytopenias with normal conventional and FISH cytogenetic studies, so her overall score would be 0.5, and she would fall into the intermediate 1 risk category. She was started on treatment for her MDS, including Erythropoietin and G-CSF.

**Figure 2:**
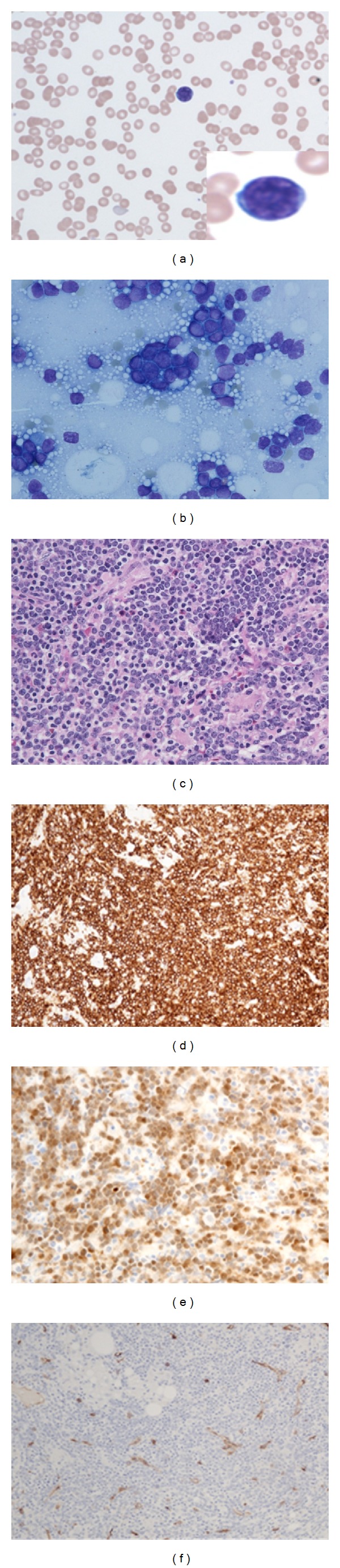
Peripheral blood smear (a) and the followup bone marrow at two months after initial presentation. Peripheral blood smear show marked pancytopenia with rare circulating lymphoblasts ((a) and inset). Bone marrow smear (b) and biopsy (c) showing sheets and clusters of lymphoblasts. These blasts show diffuse strong cytoplasmic staining for CD10 (d), diffuse nuclear staining for Terminal deoxynucleotidyl transferase (e), but were negative for CD34 (f).

Two months after the initial presentation, she was evaluated for a possible allogeneic stem cell transplantation, and her CBC revealed severe pancytopenia with WBC = 1.0 × 10^9^/L, hemoglobin = 8.7 g/dL, hematocrit = 25.1%, MCV = 82 fL, and platelets = 74.0 × 10^3^/uL. Rare circulating blasts were identified in the peripheral blood smears (Figure 2(a)). Bone marrow biopsy was then performed and showed markedly hypercellular (98%) with 82% B-lymphoblast population (Figures 2(b)–2(f)). The blasts were intermediate sized with fine chromatin, small nucleoli, nuclear folding, and scant cytoplasm. No granules or Auer rods were identified. Cytochemical stains for myeloperoxidase, Sudan black B, and dual esterase were all negative. Flow cytometric analysis showed a lymphoblast population expressing CD19, CD24, and bright CD38 at 71% of ficolled cells, consistent with precursor B-cell acute lymphoblastic leukemia. Clonality was also confirmed by a positive molecular study for immunoglobulin heavy chain gene rearrangement. Severe reticulin fibrosis was also present. A final diagnosis of precursor B-cell acute lymphoblastic leukemia (pre-B ALL) and marked reticulin fibrosis was made. Cerebrospinal fluid was negative for lymphoblasts. She was started on an induction therapy with hyper-CVAD (cyclophosphamide, vincristine, doxorubicin/adriamycin, and dexamethasone). She also received intrathecal prophylaxis (methotrexate and cytarabine) and tolerated well.

Follow-up bone marrow at 28 days after her chemotherapy showed a hypocellular marrow (20%) with panhypoplasia, persistent reticulin fibrosis, but no residual leukemic blasts were seen. Flow cytometry analysis was also negative for blasts. Patient subsequently underwent allogenic stem cell transplantation, seven months after her initial diagnosis of MDS, and five months after she developed pre-B ALL. She tolerated the procedure and was alive upon the last follow-up.

## 3. Discussion

Myelodysplastic syndromes (MDS) comprise a heterogeneous group of hematopoietic cell disorders characterized by cytopenias, bone marrow hypercellularity, and abnormal blood cell differentiation (ineffective hematopoiesis) [27]. Myelofibrosis and/or sclerosis (reticulin/collagen) can occur in wide variety of neoplastic and nonneoplastic conditions of the bone marrow disorders including MDS [28]. About 5% to 10% of patients with primary MDS and up to 50% of therapy-related MDS have significantly increased marrow reticulin fibers or even collagen fibrosis. MDS with myelofibrosis is characterized by a marked increase in bone marrow reticulin fibers and presents with pancytopenia and minimal or absent organomegaly.

The prognosis for patients with MDS with fibrosis is generally worse than that for MDS without fibrosis [29–34]; however, controversies exist [35, 36]. The variation may be secondary to the case selection in reported series. In one of the largest series of retrospective study of 352 MDS patients, the investigators reported a median survival of 9.6 months in patients with fibrosis compared to 17.4 months in those without fibrosis [29]. However, this study was performed before the development of the IPSS, so the two groups were not stratified according to other features now known to affect the survival in MDS [26]. There are other studies showing that myelofibrosis has prognostic relevance independent of the IPSS classification of MDS [31–33, 37]. Overall, patients with myelodysplastic syndrome and myelofibrosis are reported to have shorter survival times than those without these features [27–29, 37–40].

The clinical implications of increased reticulin seem to be different from those of increased collagen: the amount of bone marrow reticulin shows little correlation with the severity of the underlying hematologic disease while the presence and amount of collagen fibers are strongly correlated with abnormal blood counts and poor outcome [39]. Moreover, reticulin fibrosis is often reversible after therapeutic intervention, while collagen fibrosis is less likely to be modified by treatment. Historical observations suggested that bone marrow fibrosis might also affect hematopoietic reconstitution after allogeneic stem cell transplantation. In one study, the authors found a higher risk of graft failure and delayed neutrophil engraftment as well as a significantly higher risk of relapse in patients with severe bone marrow fibrosis compared to those with no or moderate fibrosis [39].

Approximately 20–50% of cases of MDS eventually progress to acute myelogenous leukemia, while progression of MDS into acute lymphoblastic leukemia is rare [1]. In the study of Warlick and Miller, 15% (6/41) of cases of acute leukemia transformation after primary MDS show hybrid blast phenotype with both myeloid and lymphoid markers expressed [26]. The MDS progression to pure ALL is indeed a rare phenomenon, and to date there are only 35 case reports of such progression (Summary in Table 1). This phenomenon could be explained by the fact that MDS is a disorder of the pluripotent hematopoietic stem cell. Nevertheless, Ogata et al. performed flow cytometric studies on blood and bone marrow samples from 116 patients with MDS and AML and demonstrated that a high proportion of the enriched blast cells (EBCs) from almost all MDS patients showed an immunophenotype of committed myeloid precursors (CD34+/CD38+/HLA-DR+/CD13+/CD33+) regardless of the disease subtype. They concluded that MDS EBCs often coexpressed stem cell antigens and late-stage myeloid antigens asynchronously, but rarely expressed T- and B-lymphoid cell-specific antigens [41].

Including our case, the median age of MDS patients who were reported to have later ALL transformation was 53.5 years (9–90 years). The transformation occurred between 2 and 50 months after the primary diagnosis, usually less than 2 years. A male predominance (M : F = 3 : 1) is evident. The common types of myelodysplasia associated with ALL transformation include and refractory anemia (31%, 11/36), refractory anemia with excess blasts (39%, 14/36), refractory anemia with ring sideroblasts (22%, 8/36). One case of eosinophilic MDS (2.5%), one case of chronic myelomonocytic leukemia (2.5%), and our case MDS with myelofibrosis (2.5%) have also been reported.

From the 22 cases in which the data were available, eight patients were refractory to treatment or died early during induction therapy. Ten patients achieved complete remission (CR) and 2 achieved partial remission (PR). The patients with T-ALL transformation had better prognosis than those with B-cell ALL.

## 4. Conclusion

Herein, we described a unique patient with MDS and myelofibrosis transformed to a pre-B acute lymphoblastic leukemia, which supports the hypothesis that MDS is a disorder of the pluripotent hematopoietic stem cells. However, the underlying mechanisms of lymphoid transformation are not well defined. Further studies may be necessary for better understanding of the mechanism in order to develop better management plans for these patients.

## Figures and Tables

**Table 1:** Previously reported cases of lymphoid transformation of myelodysplastic syndrome.

Serial number	Age/sex	MDS subtype	Time to progression (months)	Phenotype	Cytogenetic findings	Clinical outcome	Ref.
1	68/M	RARS	31	B cell	+8	DOD	[1]
2	9/M	RA	21	B cell	NA	Lost to Followup	[2]
3	68/F	RAEB	5	B cell	−3, 5q-	DOD	[3]
4	54/M	RA	30	B cell	Ph, 20q-	DOD	[4]
5	50/M	RA	4	B cell	Complex	CR	[5]
6	43/M	Eosinophilic MDS	11	B cell	Complex	DOD	[6]
7	46/M	RAEB	2	B cell	NA	CR	[7]
8	70/M	RA	22	B cell	NA	NA	[8]
9	68/F	RA	2	B cell	NA	NA	[9]
10	65/M	RA	18	B cell	+13	PR	[10]
11	28/M	RA	11	B cell	Normal	Refractory	[11]
12–17	65–72; 5 M, 1F	1 RA, 2RARS, 3 RAEB	4–18	T cell (All 6)	Variable	2 DOD 4 CR	[12]
18	75/M	RAEB	18	T cell	Normal	NA	[13]
19	53/M	CMML	42	T cell	Normal	PR	[14]
20	53/M	RARS	50	Null cell	NA	CR	[15]
21	90/M	RAEB	5	cALL	ND	NA	[16]
22	58/M	RA	10	L	NA	DOD	[17]
23	50/F	RAEB	3	cALL	Normal	AWD	[18]
24	67/M	RARS	24	L	Aneuploidy	CR	[19]
25	20/M	RAEB	5	T cell	NA	CR (2 yrs)	[20]
26–31	78^†^	RA 1 RARS 2 RAEB(t) 3	12	B + M 6 cases	ND	NA	[21]
32	76/M	RAEB	12	Myeloid + null	+8, +13	NA	[22]
33	72/M	RA	14	Myeloid + null	Normal	NA	[23]
34	69/F	RARS	48	Myeloid/cALL	ND	NA	[24]
35	57/F	RAEB	6	Myeloid + T	ND	NA	[25]
Our case	53/F	MDS-F	2	B cell	Normal	Post allogenic SCT; AWD	

CR: complete response; PR: partial response; RA, refractory anemia; RAEB, refractory anemia with excess blasts; RARS, refractory anemia with ring sidoblasts; CMML, chronic myelomonocytic leukemia; MDS-F, myelodysplastic syndrome with myelofibrosis; L, lymphoid lineage; B, B-cell lineage; T, T-cell lineage; cALL, common acute lymphoblastic leukemia; Myeloid, myeloid lineage; null, lymphoid progenitor without specific markers for B- or T-cell lineages; NA, not available; ND, not done; AWD, alive with disease; DOD, died of disease.

^†^Median age.
